# Using arterial blood as a substitute for venous blood in routine biochemistry parameter examinations in rabbits

**DOI:** 10.1186/s12917-020-02687-8

**Published:** 2020-11-30

**Authors:** Jia-yu Wang, Yu-bo Wang, Kun Liu, Xiao-jun Bi, Jie Sun

**Affiliations:** 1 Department of Medical Ultrasound, Tongji Hospital, Tongji Medical College, Huazhong University of Science and Technology, No. 1095 Jiefang Avenue, 430030 Wuhan, China; 2 Department of Medical Ultrasound, Maternal and Child Hospital of Hubei Province, No. 745 Wuluo Road, 430070 Wuhan, China; 3 Department of Medical Ultrasound, Jianghan University Affiliated Hubei Third People’s Hospital, No. 26 Zhongshan Avenue, 430033 Wuhan, China

**Keywords:** Arterial blood, Venous blood, Routine biochemistry parameters, Rabbits

## Abstract

**Background:**

It has been widely accepted that there is a significant difference in peripheral blood oxygen between arteries and veins. Therefore, arterial blood has been collected for blood gas analysis, and venous blood, because it is convenient to collect, has been used for most laboratory examinations. However, venous blood is always difficult to collect in rabbits; in contrast, arterial blood is easier to obtain, and research on whether arterial blood can be used instead of venous blood for routine biochemical parameter examination is rare. Therefore, the present study was designed to explore whether arterial blood can be used as a substitute for venous blood for routine biochemistry parameter examination in rabbits.

**Results:**

Three venous blood samples with gross hemolysis were excluded. Venous and arterial blood samples were obtained from forty-two rabbits. Arterial blood samples correlate well with venous blood in alkaline phosphatase (ALP), aspartate aminotransferase (AST), alanine transaminase (ALT), gamma-glutamyl transpeptidase (GGT), total protein (TP), globulin (GLB), serum total cholesterol (TC), serum triglyceride (TG), high-density lipoprotein cholesterol (HDL), low-density lipoprotein cholesterol (LDL), urea (Ur) and creatinine (Cr) levels by Deming regression analysis with slopes ranging from 0.893 to 1.176 and intercepts ranging from − 4.886 to 5.835. Bland-Altman analysis showed that the two sample parameters had 93%-98% of the points within the 95% consistency limits. There were significant differences between venous blood and arterial blood in ALP, TP, TC, TG, HDL, LDL and Cr, while AST, ALT, GGT, GLB and Ur showed no significant differences.

**Conclusions:**

Arterial blood can be a substitute for venous blood in routine biochemistry parameter examinations in rabbits, especially in situations where venous blood is difficult to collect.

## Background

Rabbit models of different diseases have been widely used in animal experiments. Therefore, blood sample collection from rabbits for biochemical assays is also widely performed throughout entire experiments. Venous blood sampling from rabbits for most biochemical assays is performed at the ear marginal vein [1]; thus, venous blood is difficult to collect because the ear vein is small and nonelastic, making the vein wall coherent when pumped by an injection syringe or vacuum tube [2]. Venous blood flows into the vacuum tube so slowly that the earliest blood in the tube may clot, which is unsuitable for biochemical analysis. An unsedated rabbit will often shake its head and dislodge the needle from its ear, creating a large hematoma and making repeated sampling from the same ear difficult [3]. The targeted vein always has a thrombus after the venous indwelling needle is removed, which makes repeated collection of venous blood difficult. Otherwise, hemolysis easily occurs in rabbit venous blood collection with the use of small-gauge needles and small and fragile veins that are easily traumatized [4].

Previous research has tried to collect venous blood by jugular puncture; however, this procedure can also stimulate the vagus nerve, which can cause heart arrhythmias ranging in severity from bradycardia to complete sinoatrial or atrioventricular block [3]. Arterial puncture for humans is more time consuming and more painful and may lead to more complications than venous puncture [5–7]. However, the central artery of the rabbit ear is different from that of humans; it is as superficial as the ear vein and more elastic than veins, which makes it unable to collapse during puncture. Therefore, arterial blood collection from rabbits is easier than venous blood collection and adequate for repeating sampling. Using arterial blood instead of venous blood from rabbits for biochemical assays may provide convenience for the experiment. Unfortunately, studies on biochemical parameter differences between arterial and venous blood are rare. Our study was designed to explore the consistency between venous and arterial blood in some routine laboratory examinations in rabbits and to determine whether arterial blood can be a substitute for biochemical assays when venous blood is difficult to collect.

## Results

Routine biochemical parameters were assessed for all 45 rabbits. Three venous blood samples showed visible hemolysis and were excluded. A total of 42 rabbits were included.

**Fig. 1:**
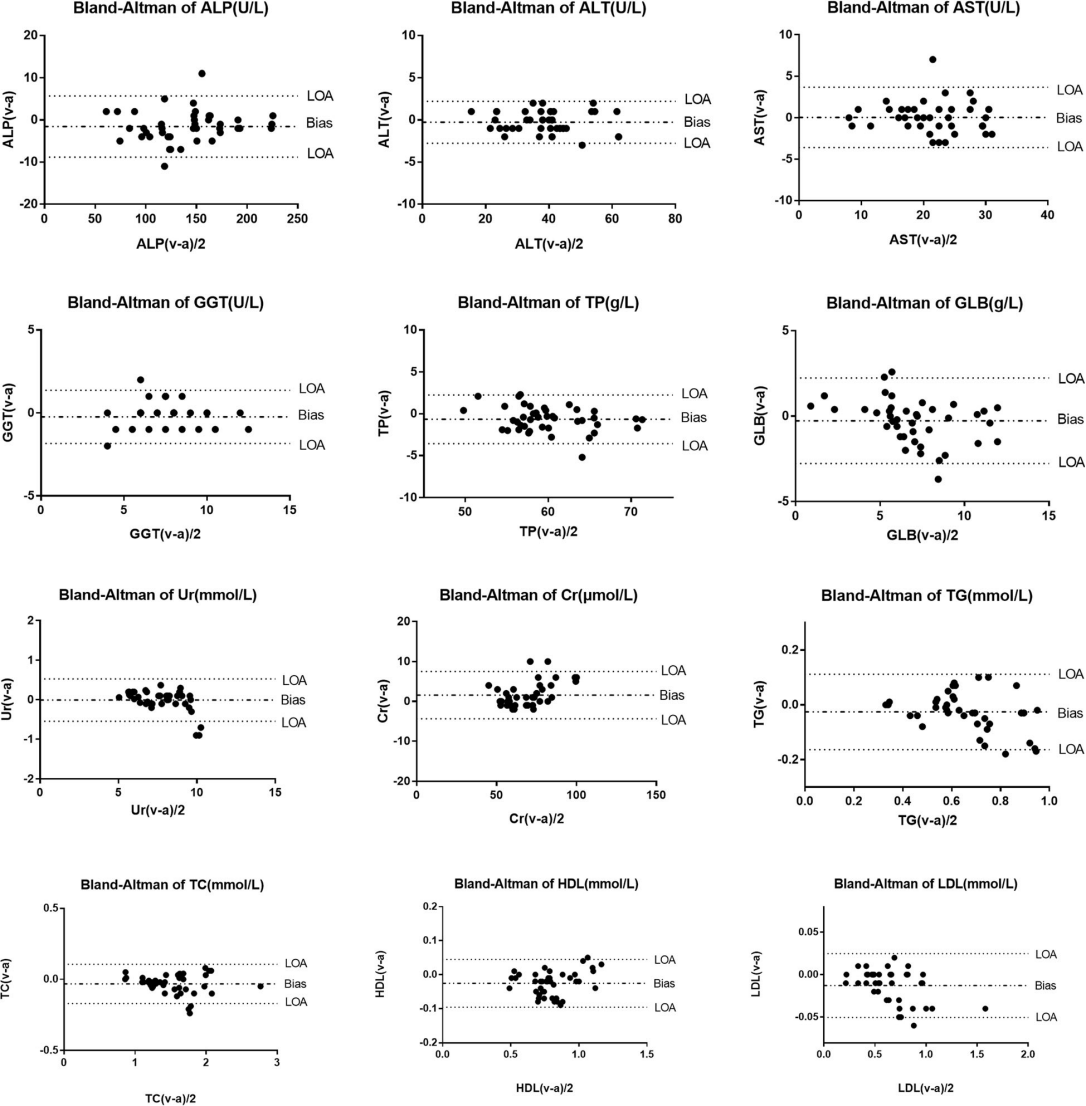
Bland-Altman analysis of routine biochemical parameters of venous and arterial blood samples

**Fig. 2:**
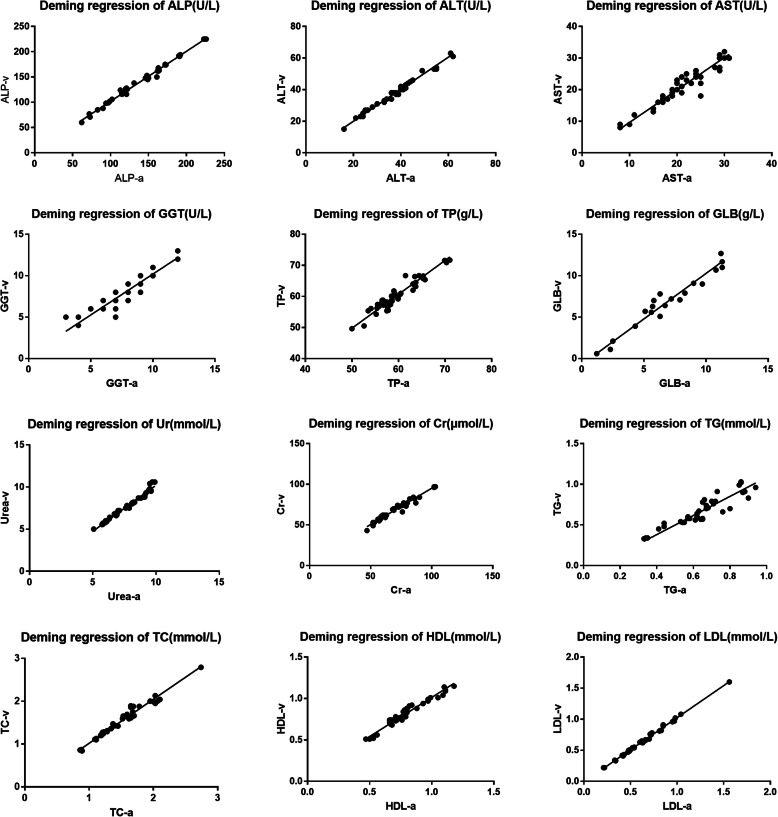
Deming regression analysis of routine biochemical parameters of venous and arterial blood samples

Routine biochemical parameters of venous and arterial blood samples and statistical parameters are listed in Table 1. There were significant differences between venous blood and arterial blood in ALP, TP, TC, TG, HDL-C, LDL-C and Cr, while AST, ALT, GGT, GLB and urea showed no significant differences. ALT, AST, Cr, HDL and LDL had 98% of the points within the 95% consistency limit. TP, ALP, GGT, urea and TG had 95% of the points within the 95% consistency limit. GLB and TC had 93% of the points within the 95% consistency limit (Fig. 1). The Deming regression analysis showed that all the slopes were near one (0.893–1.176) and that the intercepts except ALP (3.147), TP (-4.886) and Cr (5.835) were small (Fig. 2). The 95% confidence intervals of slopes didn’t contain 1 in Ur, Cr, TG and LDL. Meanwhile, the 95% confidence intervals of intercepts didn’t contain 0 in Ur, Cr and HDL (Table 1).

**Table 1:** Routine biochemical parameters of venous and arterial blood samples in rabbits

	Venous blood	Arterial blood	*P*	Bias	Slope (95% confidence intervals)	Intercept (95% confidence intervals)
ALP (U/L)	140.3 ± 40.72	138.70 ± 41.19	0.010	-1.548	0.989 (0.960 to 1.017)	3.147 (-0.969 to 7.262)
ALT (U/L)	39.00 ± 11.12	38.74 ± 11.00	0.189	-0.262	1.011 (0.974 to 1.049)	-0.183 (-1.676 to 1.310)
AST (U/L)	21.74 ± 6.54	21.79 ± 6.38	0.869	0.048	1.026 (0.929 to 1.123)	-0.620 (-2.824 to 1.584)
GGT (U/L)	7.595 ± 1.89	7.36 ± 1.91	0.067	-0.238	0.987 (0.840 to 1.134)	0.334 (-0.780 to 1.449)
TP (g/L)	60.33 ± 5.11	59.67 ± 4.69	0.007	-0.655	1.093 (0.988 to 1.198)	-4.886 (-11.180 to 1.411)
GLB (g/L)	6.90 ± 3.34	6.93 ± 3.04	0.889	0.025	1.099 (0.972 to 1.227)	-0.714 (-1.677 to 0.249)
Ur (mmol/L)	7.77 ± 1.51	7.77 ± 1.38	0.204	-0.006	1.096 (1.037 to 1.155)	-0.742 (-1.205 to -0.278)
Cr (µmol/L)	68.07 ± 12.94	69.67 ± 14.45	0.002	1.595	0.893 (0.838 to 0.949)	5.835 (1.886 to 9.785)
TC (mmol/L)	1.544 ± 0.3925	1.512 ± 0.3818	0.009	-0.032	1.028 (0.969 to 1.088)	0.011 (-0.104 to 0.083)
TG (mmol/L)	0.6833 ± 0.1841	0.6571 ± 0.1585	0.020	-0.026	1.176 (1.023 to 1.328)	-0.089 (-0.192 to 0.014)
HDL (mmol/L)	0.8174 ± 0.1651	0.7919 ± 0.1746	< 0.001	-0.025	0.945 (0.882 to 1.007)	0.069 (0.019 to 0.120)
LDL (mmol/L)	0.6374 ± 0.2756	0.6245 ± 0.2667	< 0.001	-0.013	1.033 (1.013 to 1.054)	-0.008 (-0.022 to 0.006)

Data were analyzed by *p* value of paired t-test or Wilcoxon test, bias of Bland‐Altman analysis, slope and intercept of Deming regression

## Discussion

Venous blood is widely used in clinical diagnosis and evaluation; however, venous puncture is difficult to perform, especially in some small experimental animals, such as rabbits and mice. The veins are small and friable, the blood collection procedure does not always go smoothly, and forced extrusion of the vein causes hemolysis to occur. Therefore, venous blood is difficult to collect for laboratory analysis, whereas arterial blood is more convenient to obtain in small animals. It is well known that venous blood and arterial blood are different in regard to oxygen and carbon dioxide levels; therefore, blood gas analysis was performed only on arterial blood [8]. Other studies also showed that the results from a complete blood count obtained from canine and postpubertal rabbit venous and arterial blood samples may not be comparable [9, 10]. However, studies on whether arterial blood can be used instead of venous blood for hepatic function, renal function and serum lipid levels in rabbits are rare.

In our study, we used healthy rabbits as a model, analyzing the differences in some common biochemical assay parameters between arterial blood and venous blood. We found that all of the parameters showed excellent consistency by Bland-Altman analysis, and the two samples correlated well. The results of Deming regression analysis indicated that most of the parameters demonstrated excellent consistency between arterial blood and venous blood. However, it can be considered that the proportional deviation was existed in Ur, Cr, TG and LDL between venous blood and arterial blood via the analysis of 95% confidence intervals of slopes. Whereas constant deviation was found in Ur, Cr and HDL between venous blood and arterial blood via the analysis of intercepts. Moreover, we noticed that there are significant differences between venous blood and arterial blood in ALP, TP, Cr and some blood lipid parameters, including TC, TG, HDL, LDL, which may be partly caused by hemolysis of the venous blood, which is unavoidable when collecting venous blood from rabbits. Although samples with gross hemolysis were excluded from our study, there is also a discharge of the cell constituents into serum or plasma due to hemolysis undetectable by visual inspection [11]. Previous clinical research has shown that hemolysis affects the plasma concentration of a whole range of biochemical parameters, whereas the most prominent effect of hemolysis is observed for AST, lactate dehydrogenase, potassium and total bilirubin. The differences in ALP, HDL, TP and some other analytes were statistically significant but remained within Clinical Laboratory Improvement Amendments (CLIA) limits [12]. Giuseppe Lippi reported that hemolysis generated a consistent trend towards overestimation of ALT, AST, Cr and Ur [13]. A study showed that hemolysis interference was detected for ALP, AST, GGT, TP, ALT and other analytes that were not mentioned in our study [14]. From the above studies, we found that different studies obtained different parameters that were affected by hemolysis. In our study, ALP and TP were higher in venous blood, while AST and ALT showed no difference, which may be caused by hemolysis; for experiments performed on different species, the results may be partly different. For a higher Cr in arterial blood, we think that this is a normal phenomenon caused by filtering by the kidneys and that part of the metabolic substance was excreted with urine. Ur in arterial blood should be higher than that in venous blood, but our study showed that there are no differences between the two samples; furthermore, there may be an overestimation of venous blood Ur due to venous blood hemolysis. A previous study showed that Ur is elevated with moderate hemolysis, even at the greatest degree of hemolysis, and no interference was detected for Cr [14]. This is coincident with our study. However, the biases of ALP, TP, Cr and blood lipids were subtle and not deemed clinically important; therefore, we considered arterial blood instead of venous blood for routine biochemical parameters in rabbits.

A previous study has shown that arterial and venous blood can be used interchangeably to study the effect of blood concentrations of common soluble surrogate markers of atherosclerosis in humans, and there are virtually the same LDL, HDL, TC and TG levels in arterial plasma compared to venous plasma [15, 16]. In our study, we found that in rabbits, all the lipid parameters of the venous sample were higher than those of the arterial sample, which may be caused by venous blood hemolysis that is undetectable by visual inspection, as explained above. Whereas the mean differences are subtle, the biases between lipid parameters of the two samples are subtle, and the agreements of lipid parameters between the two samples are good. The correlations between lipid parameters of the two samples by Deming regression were good; therefore, we think that in rabbits, arterial blood can be used for rough estimation of the lipid level while taking the magnitude of bias into account.

### Limitation

Our study was limited by the relatively small sample size and simple laboratory parameters. A larger sample size and more study parameters are needed for a more accurate and comprehensive result. Moreover, in our study, we excluded only gross hemolysis and did not evaluate the degree of hemolysis; therefore, the reasons for the higher venous value of ALP, TP and blood lipid parameters were based entirely on supposition. Studies on the influence of hemolysis on rabbit venous blood may provide an exact result.

Importantly, venous blood collection cannot avoid hemolysis, so we focused on using arterial blood instead of venous blood, even though the samples have invisible hemolysis, which may provide more accurate biochemical parameters.

## Conclusions

Our study has shown that most of the parameters showed no significant difference between two blood samples and that although some parameters showed statistically significant differences, the biases were subtle and were not deemed clinically important. The two samples correlated well and had excellent consistency, which implied that arterial blood can be a potential surrogate of venous blood in biochemical assays in rabbits.

## Methods

This study was approved by the Institutional Animal Care and Use Committee of Tongji Hospital (No. 478). Forty-five healthy male New Zealand rabbits weighing 1.8 kg to 2.5 kg were included in this study. They were obtained commercially from the experimental animal center of Hubei Province. All rabbits were fed in separate cages in a standard environment. Heparinized vacuum test tubes were used for blood collection. The animals had been fasted for 12 hours but allowed free access to water before the experiments. Venous blood was obtained by rabbit auricular vein puncture. When collecting, the blood collection needle was fixed, and the vacuum tube was rotated intermittently to prevent clotting. Arterial blood was obtained by rabbit ear central artery puncture immediately after venous blood collection. At least 5 milliliters of blood was collected in each tube. Samples with visible hemolysis were excluded. The remaining samples were sent to the laboratory for testing within one hour. All the samples were centrifuged by a Baiyang centrifuge machine (Instrumentation Laboratory, Beijing, China) at 3000 r/min for 10 minutes. Taking the supernatant, an automated biochemistry analyzer (Roche Cobas 8000 c701) was used for analysis of enzymes including alkaline phosphatase (ALP), aspartate aminotransferase (AST), alanine transaminase (ALT), gamma-glutamyl transpeptidase (GGT). Hepatic functional parameters include total protein (TP) and globulin (GLB). Serum lipid parameters include serum total cholesterol (TC), serum triglyceride (TG), high-density lipoprotein cholesterol (HDL) and low-density lipoprotein cholesterol (LDL). Renal function parameters include urea (Ur) and creatinine (Cr). All rabbits were euthanized by intravenous injection with 3% pentobarbital after the study.

### Statistical analysis

Data analysis was performed with GraphPad Prism software (GraphPad software, La Jolla, CA, USA). All values are expressed as the mean ± standard deviation. The differences between venous and arterial parameters that met a normal distribution were analyzed by paired *t* tests, and parameters that did not meet a normal distribution were analyzed by Wilcoxon tests. The consistency between venous blood and arterial blood was assessed with Bland-Altman analysis. Bias was defined as the mean value of the difference between the paired venous and arterial blood parameters. Limits of agreement were defined as  A Deming regression analysis was used to determine the correlation between venous and arterial blood samples. A *p* value < 0.05 was statistically significant.

## Data Availability

The datasets used and/or analyzed during the current study are available from the corresponding author on reasonable request.

## Abbreviations

ALP: Alkaline phosphatase
AST: Aspartate aminotransferase
ALT: Alanine transaminase
GGT: Gamma-glutamyl transpeptidase
TP: Total protei
GLB: Globulin
TC: Total cholesterol
TG: Serum triglyceride
HDL: High-density lipoprotein cholesterol
LDL: Low-density lipoprotein cholesterol
Ur: Urea
Cr: Creatinine
